# The metagenome of the female upper reproductive tract

**DOI:** 10.1093/gigascience/giy107

**Published:** 2018-09-06

**Authors:** Fei Li, Chen Chen, Weixia Wei, Zirong Wang, Juanjuan Dai, Lilan Hao, Liju Song, Xiaowei Zhang, Liping Zeng, Hui Du, Huiru Tang, Na Liu, Huanming Yang, Jian Wang, Lise Madsen, Susanne Brix, Karsten Kristiansen, Xun Xu, Junhua Li, Ruifang Wu, Huijue Jia

**Affiliations:** 1 BGI Education Center, University of Chinese Academy of Sciences, Shenzhen 518083, China; 2 BGI-Shenzhen, Shenzhen 518083, China; 3 China National GeneBank, BGI-Shenzhen, Shenzhen 518120, China; 4 Peking University Shenzhen Hospital, Shenzhen 518036, China; 5 Shenzhen Key Laboratory on Technology for Early Diagnosis of Major Gynecological diseases, Shenzhen, PR China; 6 BGI genomics, BGI-Shenzhen, Shenzhen 518083, China; 7 Laboratory of Genomics and Molecular Biomedicine, Department of Biology, University of Copenhagen, Universitetsparken 13, 2100 Copenhagen, Denmark; 8 Shenzhen Key Laboratory of Human Commensal Microorganisms and Health Research, BGI-Shenzhen, Shenzhen 518083, China; 9 James D. Watson Institute of Genome Sciences, Hangzhou310000, China; 10 Macau University of Science and Technology, Taipa, Macau 999078, China; 11 Institute of Marine Research (IMR), Postboks 1870, Nordnes, N-5817, Bergen, Norway; 12 Department of Biotechnology and Biomedicine, Technical University of Denmark, Soltofts Plads, 2800 Kongens. Lyngby, Denmark; 13 School of Bioscience and Biotechnology, South China University of Technology, Guangzhou 510006, China

**Keywords:** metagenomics, microbiota, female upper reproductive tract

## Abstract

**Background:**

The human uterus is traditionally believed to be sterile, while the vaginal microbiota plays an important role in fending off pathogens. Emerging evidence demonstrates the presence of bacteria beyond the vagina. However, a microbiome-wide metagenomic analysis characterizing the diverse microbial communities has been lacking.

**Results:**

We performed shotgun-sequencing of 52 samples from the cervical canal and the peritoneal fluid of Chinese women of reproductive age using the Illumina platform. Direct annotation of sequencing reads identified the taxonomy of bacteria, archaea, fungi and viruses, confirming and extending the results from our previous study. We replicated our previous findings in another 24 samples from the vagina, the cervical canal, the uterus and the peritoneal fluid using the BGISEQ-500 platform revealing that microorganisms in the samples from the same individuals were largely shared in the entire reproductive tract. Human sequences made up more than 99% of the 20GB raw data. After filtering, vaginal microorganisms were well covered in the generated reproductive tract gene catalogue, while the more diverse upper reproductive tract microbiota would require greater depth of sequencing and more samples to meet the full coverage scale.

**Conclusions:**

We provide novel detailed data on the microbial composition of a largely unchartered body site, the female reproductive tract. Our results indicated the presence of an intra-individual continuum of microorganisms that gradually changed from the vagina to the peritoneal fluid. This study also provides a framework for understanding the implications of the composition and functional potential of the distinct microbial ecosystems of the female reproductive tract in relation to health and disease.

## Background

Evolution of the female reproductive tract has resulted in complex and unique structures such as the uterus, cervix and the vagina. The human vagina hosts trillions of bacteria that can significantly impact the health of women and their neonates. The cervix has traditionally been regarded to function as a perfect barrier between the vagina and uterus leading to the assumption that the upper reproductive tract constitutes a sterile environment. However, judging from evidence in insects and other animals, humans are probably no exception with regard to possible vertical transmission of the mothers’ microbiota before birth [1]. Thus, in humans, bacterial DNA has been detected in the placenta [2, 3]. Based on our recent analyses using 16S rRNA gene amplicon sequencing, the upper reproductive tract, including cervix, uterus, fallopian tubes, and peritoneal fluid, harbors diverse communities of bacteria, though at low abundance [4].

Recent studies of female reproductive tract microbiota have mainly focused on the vagina using 16S rRNA gene amplicon sequencing [5–7]. Studies using 16S rRNA gene amplicon sequencing have limitations in relation to lower taxonomic resolution and the lack of ability to perform species-specific functional inference. Metagenomic shotgun sequencing can address these limitations, but only a few studies have applied metagenomic shotgun sequencing to the vaginal microbiota [8], and no studies have characterized the compositional range of the upper reproductive tract microbiome using metagenomic analysis. The present study is the first to provide metagenomic data from the female upper reproductive tract.

## Data Description

Samples of six locations (CL, lower third of vagina; CU, posterior fornix; CV, cervical mucus drawn from the cervical canal; ET, endometrium; FLL and FRL, left and right fallopian tubes; and PF, peritoneal fluid from the pouch of Douglas) throughout the female reproductive tract from 137 Chinese women of reproductive age undergoing surgery for conditions not known to involve infection (Supplementary Table S1) were collected for this study. The 16S rRNA gene amplicon sequencing was performed on 665 of these samples. The results from 476 samples have been published previously [4], and results from the remaining 189 are presented in this study. Two samples (1 CV and 1 CU) were subjected to shotgun sequencing with or without prior removal of human DNA using a commercial kit to test the experimental effect of removal of host DNA before sequencing (refer to the Methods section). Then, 25 PF and 25 CV samples were sequenced on the Illumina HiSeq platform using 100 bp paired-end (PE) sequencing (for the stringent selection rules of samples, see the Methods section for details). For these 52 samples, 20 GB of raw data per sample, corresponding to 0.99 TB, were generated. Additionally, intra-individual similarity in the vagino-uterine microbiota was also examined based on 24 samples from different sites of the reproductive tract (CL, CU, CV, ET, PF) in six women. These samples were sequenced on the BGISEQ-500 sequencer using 100 bp single-end (SE) sequencing; 60 GB of raw data per sample were generated, totaling 1.40 TB. The dataset after filtering out low-quality and host reads (refer to this Methods section) is available at the European Bioinformatics Institute (EBI) database using the accession number PRJEB24147.

## Analyses and Discussion

### Metagenomic sequencing

According to shotgun sequencing of vaginal samples in the Human Microbiome Project and of placental samples by Aagaard et al., more than 90% of the sequences were derived from human host DNA [2, 9]. To overcome this problem, we first tested a commercial kit that removes human DNA by binding and precipitating CpG-methylated DNA. Unfortunately, after the kit treatment, a considerable amount (99.9% for CV sample and 79% for CU sample) of host DNA still remained (Supplementary Fig. S1a). Furthermore, the bacterial composition was altered following kit treatment when compared with the control group (Supplementary Fig. S1b). We therefore abandoned the strategy of host DNA removal prior to shotgun metagenomics sequencing.

The sample selection was based on the data from CV and PF samples [4], which we identified as robust representations of the overall samples. Since higher amounts of DNA is required for shotgun-sequencing compared to 16S rRNA gene amplicon sequencing, more stringent criteria for selection of samples were applied including individual sub-clusters representation and sufficient DNA amounts (see details in the Methods section). According to the cluster criterion, clustering results based on the relative abundance of operational taxonomic units (OTUs) in the PF and CV samples showed that the samples marked with red (all containing DNA >1 μg) were well distributed among all collected samples (Supplementary Fig. S2), and therefore selected for shotgun sequencing in this study. Hence, 25 PF and 25 CV samples were selected for sequencing using the Illumina HiSeq 4000 platform. After quality control, high-quality reads were aligned to hg 19 using SOAP and GRCh38 using DeconSeq to remove human reads (see details in the Methods section). We observed an average host contamination of 99.72% for CV and 99.93% for PF samples (Supplementary Table S2), which are comparable to that previously reported for placenta samples [2].

Our results were further expanded by inclusion of an additional 24 samples subjected to sequencing on the BGISEQ-500 platform, in which we also examined the intra-individual similarity in the vagino-uterine microbiota based on samples from different sites of the reproductive tract (CL, CU, CV, ET, PF). The average host contamination rate for vagina (CL, CU) samples was 96.55%, and lower than those of the CV, ET and PF samples, which were all above 99.5% (Supplementary Table S2).

### A diverse microbiome in the cervical canal and the peritoneal fluid of reproductive age women

**Figure 1:**
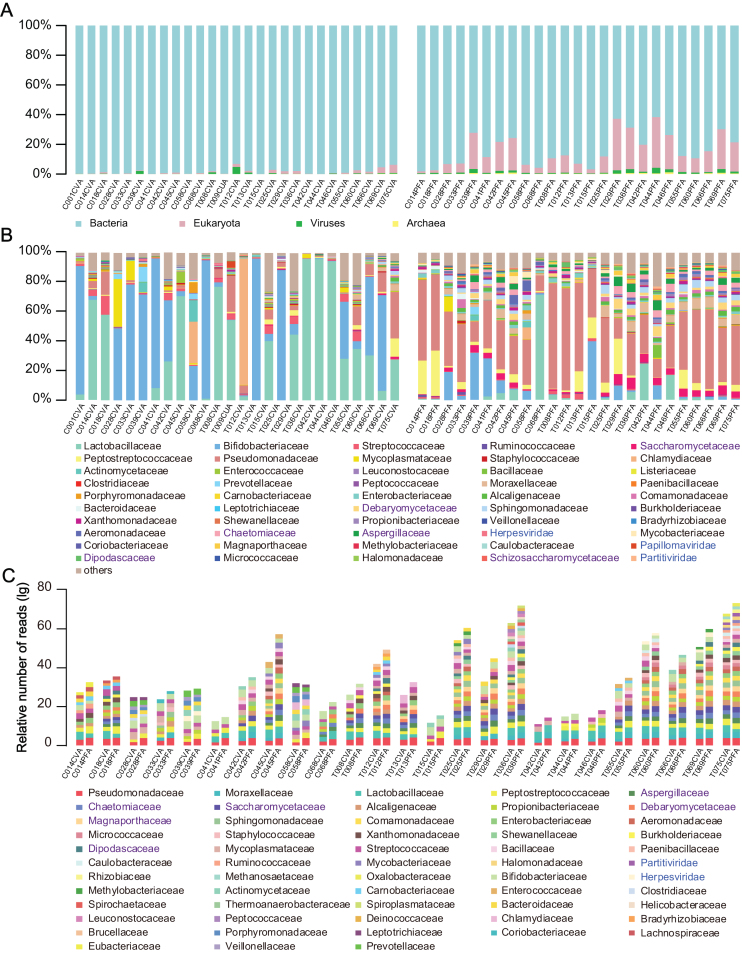
The overall microbiome composition of the cervical canal and the peritoneal fluid of reproductive-age women. Cumulative bar charts of the main taxa at domain **(A)** and family **(B)** levels in CV and PF samples. **(C)** Compositional overlap at the family level of CV and PF samples from the same individuals. Relative number of reads was calculated as ${N_p} = \frac{{{a_p}}}{{{a_t}}}\ \times m$, where ${a_p}$ is the number of reads within $p$ taxa in $a$ sample, $\ {a_t}$ is the total number of reads within $a$ sample, and *m* is the median number of reads within all 50 samples. When $p$ taxa is shared by CV and PF samples from the same individuals and at the same time, both ${N_p}\ $ values are higher than 0.1%${\rm{\ }} \times m$, the $p$ taxa is included in the cumulative bar charts. Taxa names **(B, C )** in black, purple, and blue denote bacteria, eukaryotes, and viruses, respectively.

To obtain an overview of the overall composition of the vagino-uterine microbiome, we used Kraken to directly assign sequencing reads to all types of microbial taxa [10]. The dominant *Lactobacillus* spp. in CV and *Pseudomonas* spp. in PF were detected in the present study in accordance with those found in the previous study [4]. In addition, methane-producing archaea, yeasts, herpesviruses, papillomaviruses, and bacteriophages were also identified (Fig. 1A, 1B).

The abundance of these taxonomic units varied among samples, and those constituting more than 0.1% of the total reads number and identified in the CV and PF samples from the same individual are shown in Fig. 1C.

**Figure 2:**
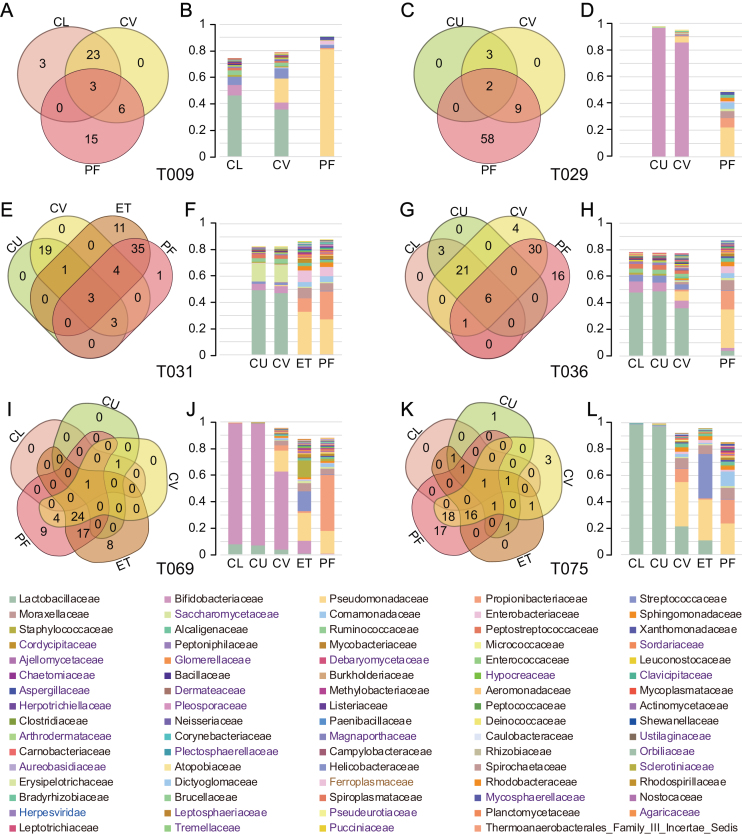
Composition of the vagino-uterine microbiota. **(A, C, E, G, I, K)** Venn diagrams depicting shared taxa at the family level in samples collected at different sites in the same individual. **(B, D, F, H, K, L)** Cumulative bar charts of the taxa with relative abundance higher than 0.1% and present in at least two sites of the same individual. Taxa names **(B, C)** in black, purple, blue, and gray denote bacteria, eukaryotes, viruses, and archaea, respectively.

To gain further insight into the compositional similarities of the microbiota at different sites of the reproductive tract in the same individual, we selected taxa at the family level that fulfilled two criteria: they were present in at least two sites of the same individual and the relative abundance was higher than 0.1%. Taxa fulfilling these criteria represented more than 45% of the microorganisms presented in the samples across the six individuals subjected for this detailed analysis (Fig. 2). *Lactobacillaceae* or *Bifidobacteriaceae* dominated in the vagina (CL and CU) but not in the upper reproductive tract, where microorganisms such as *Pseudomonadaceae, Propionibacteriaceae, Streptococcaceae*, and *Moraxellaceae* constituted a notable fraction of the microbiota. In addition, eukaryotes, viruses, and archaea, such as *Saccharomycetaceae, Herpesviridae*, and *Ferroplasmaceae*, were also found in the female reproductive tract. The results at the bacterial level are in keeping with our findings in a recent study [4], and the current data further demonstrate an intra-individual continuum of all types of microorganisms that gradually changes from the vagina to the peritoneal fluid.

### Genes from the vagino-uterine microbiota

Reference gene catalogs, especially the human gut microbiome, have greatly facilitated analyses of the microbiome[11–13]. Here, we established the first gene catalog of the microbiome of the female upper reproductive tract comprising of 60,699 genes.

**Figure 3:**
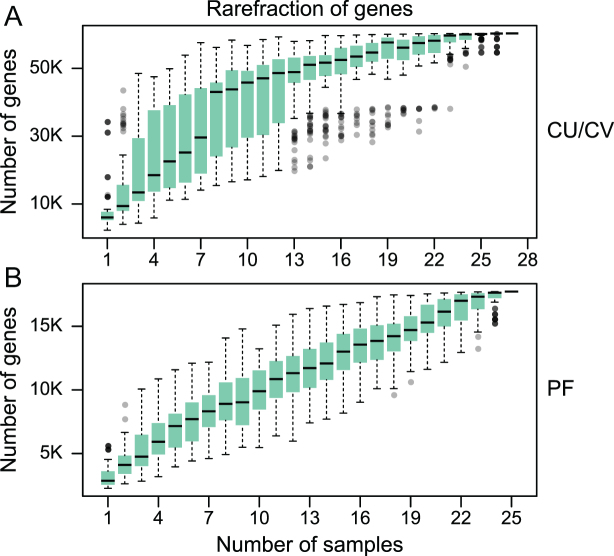
Rarefaction of microbial gene content in CV **(A)** and PF **(B)** samples. The number of genes in each group was calculated after 100 random samplings with replacement. Boxes denote the interquartile range (IQR) between the first and third quartiles (25th and 75th percentiles, respectively), and the line inside denotes the median. Whiskers denote the lowest and highest values within 1.5 times IQR from the first and third quartiles, respectively. Circles denote outliers beyond the whiskers.

Rarefaction analysis based on gene number revealed a curve approaching saturation with about 23 CV samples (Fig. 3). However, rarefaction analysis based on gene numbers in PF samples revealed a curve that leveled off, but still did not reach a plateau, possibly due to a more diverse microbiota in the PF. Therefore, with 20GB sequences per sample, vaginal bacteria were well covered, whereas a more comprehensive characterization of bacteria from the upper reproductive tract would require a higher sequencing depth and more samples.

**Figure 4:**
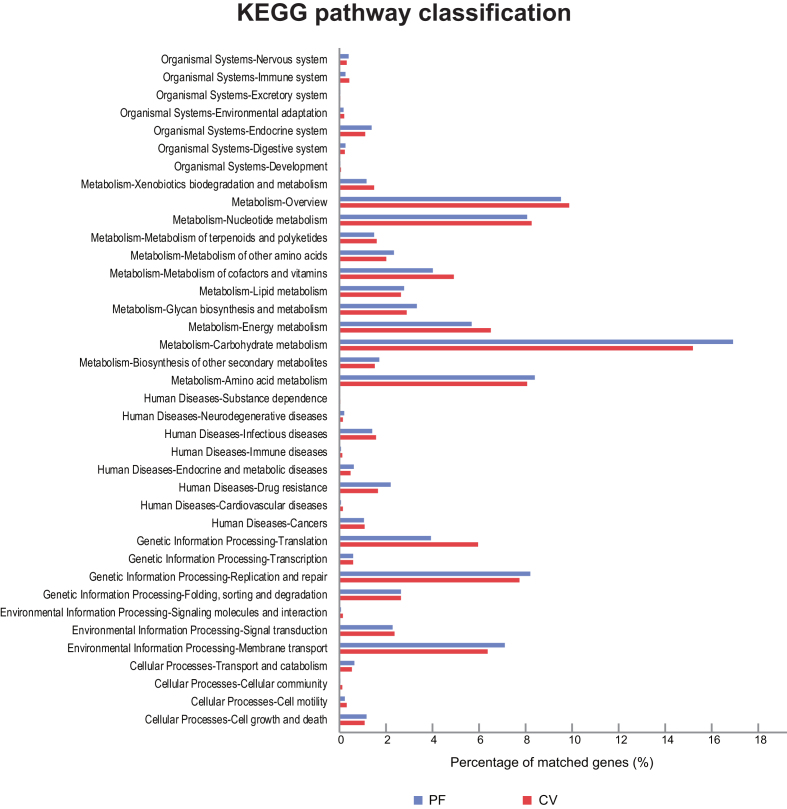
KEGG pathway classification of the vagino-uterine microbiome. Comparison of CV (red) and PF (blue) data based on KEGG annotation, which emphasizes functional similarity of the CV and PF microbiota.

We annotated the genes in the gene catalog according to the Kyoto Encyclopedia of Genes and Genomes (KEGG) [14]. The matched genes in the PF samples (15,316 genes) were all covered within the CV samples (39,087 genes). Comparing the CV and the PF samples in the distribution of KEGG pathways, the PF samples showed a greater proportion of genes involved in carbohydrate metabolism, replication and repair, membrane transport, and drug resistance, whereas genes involved in translation, energy metabolism, and metabolism of cofactors and vitamins were enriched in the CV samples (Fig. 4). In relation to KEGG orthology (KO) modules, CV samples showed enrichment of transport systems for thiamine, cystine, teichoic acid, taurine, and putative ABC transport systems compared to the PF samples. Regulatory systems of aerobic and anaerobic respiration, osmotic stress response and multicellular behavior control were also enriched in the CV samples (Supplementary Table S3).

## Methods

### Sample description

A total of 137 Chinese women of reproductive age undergoing surgery for conditions not known to involve infection (hysteromyoma, adenomyosis, endometriosis, and salpingemphraxis) were enrolled in this study (Supplementary Table S1). Samples were taken from the CL, CU, and CV on the day of the clinical visit without any prior disturbance. Depending on the clinical conditions, laparoscopy or laparotomy was performed, and samples from the ET, FLL, FRL, and PF were taken during surgery (Supplementary Table S1). The study was approved by the institutional review boards at Peking University Shenzhen Hospital and BGI-Shenzhen, and all women provided written informed consent. The subject exclusion criteria, sampling, and DNA extraction methods can be found in [4].

To test the effect of experimental removal of human DNA, one CU sample and one CV sample were used for shotgun sequencing on the Illumina HiSeq2000 platform with or without prior removal of human DNA. The NEBNext Microbiome DNA Enrichment Kit was used according to the manufacturer's instructions, with 10 μg input DNA per sample.

We performed a prior selection of samples to undergo shotgun-sequencing. The selection was based on the data from CV and PF samples [4] using the following two criteria: samples should represent individual subclusters when subjected to hierarchical (centroid-linkage) clustering based on relative abundances of OTUs from 16S rRNA gene amplicon sequencing and the amount of DNA should be greater than 1 μg. The samples with good scattering in different clusters based on the relative abundance of OTUs in the PF and CV samples were selected for shotgun sequencing on the Illumina HiSeq4000 platform.

We replicated the findings in 24 additional samples on the BGISEQ-500 platform, where additional sites (CL, CU, CV, ET, and PF) of six women were analyzed. To meet the need of library construction, the amount of DNA in all 24 samples was greater than 1 μg. Three qualified samples from each woman were set as a threshold.

### Metagenomic shotgun sequencing

Library construction and shotgun sequencing using the Illumina HiSeq2000/4000 platforms (insert size 350 bp; 100 bp of PE reads; two replicate libraries were constructed for each lane) and BGISEQ-500 (100 bp of SE reads; one library was constructed for each lane) were performed as previously described [15] (and see protocol in protocols.io [16]). The quality control of sequencing data from the HiSeq and BGISEQ platforms was also performed as previously described [15]. Human sequences were eliminated by alignment to the hg19 reference genome using SOAP2.22 (SOAPaligner/soap2, RRID:SCR_005503). As the resulting data still contained human sequences, a more stringent procedure using DeconSeq by aligning data to the GRCh38 reference genome was applied [17].

### Taxonomic assignment of sequencing reads

High-quality, non-human sequences were tentatively assigned to microbial taxa using Kraken using default parameters (Kraken, RRID:SCR_005484) [10]. For pair-end reads Kraken automatically concatenated the pairs together with a single N between the sequences using default parameters, and according to the manual this software raises the sensitivity by about 3 percentage points over classifying the sequences as single-end reads.

### Construction of a gene catalog

The high-quality, non-human sequencing reads of the 52 samples sequenced using the Illumina HiSeq platform were *de novo* assembled into contigs using IDBA-UD (IDBA-UD (RRID:SCR_011912))[18]. We used the same strategy as describe in previous studies [12, 13], where genes were predicted from the contigs by MetaGeneMark [19], and highly similar genes (95% identity, 90% overlap) were removed as redundant using CD-HIT (CD-HIT, RRID:SCR_007105) [20]. Functional annotations were made by BLASTP (v2.2.24) based on the KEGG (v76) databases (KEGG, RRID:SCR_012773)[14].

### Availability of supporting data

The sequencing data after filtering out low-quality and host reads are available via the EBI database using the accession number PRJEB24147. Additional supporting data are available via the *GigaScience* GigaDB database [21].

## Additional files

**Supplementary Figure S1:** Evaluation of the NEBNext Microbiome DNA Enrichment Kit by two comparative strategies. Sample names suffixed by “-HR” represent DNA samples that were treated with the kit for removal of host DNA before shotgun sequencing, while sample names suffixed by A represent DNA samples that were subjected to shotgun sequencing directly (**a**). The table data shows the obtained read number, and remaining reads after removal of host DNA reads in the two samples. **b**) Influence of host DNA presence on bacterial DNA identification during shotgun sequencing. The plots display the compositional difference amongst major bacteria genera in samples with and without (-HR) host DNA presence. Data were analyzed by mapping reads to the ICG bacterial reference gene catalog [12].

**Supplementary Figure S2:** Samples selected for metagenomic sequencing. Hierarchical clustering of CV (**a**) and PF (**b**) samples based on the relative abundances of OTUs. Samples which represent individual sub-clusters and hold DNA amounts above 1 μg were selected for shotgun-sequencing (red).

**Supplementary Table S1:** Phenotypic information for the 137 subjects.

**Supplementary Table S2:** Statistics for each shotgun-sequenced sample.

**Supplementary Table S3:** The distribution of the Modules in the female reproductive tract.

## Abbreviations

CL: lower third of vagina; CU: posterior fornix; CV: cervical mucus drawn from the cervical canal; ET: endometrium; FLL: left fallopian tube; FRL: right fallopian tube; KEGG: Kyoto Encyclopedia of Genes and Genomes; OTU: operational taxonomic units; PE: paired-end; PF: peritoneal fluid from the pouch of Douglas; SE: single-end.

## Competing interests

The authors declare that they have no competing interests.

## Funding

The study was supported by the Shenzhen Municipal Government of China (JCYJ20160229172757249, JCYJ20150601090833370) and a grant from the Macau Technology Development Fund (102/2016/A3).

## Author contributions

H.J. and R.W. conceived and directed the project. W.W., J.D., L.Z., H.D., H.T., and R.W. performed the clinical diagnosis and sample collection. F.L., C.C., Z.W., and L.H. performed the bioinformatic analyses and prepared display items. C.C., F.L., Z.W., X.Z., J.L., and H.J. wrote the first version of the manuscript. L.M., S.B., and K.K. revised the manuscript. All authors contributed to the final revision of the manuscript

## Supplementary Material

GIGA-D-18-00184_Original_Submission.pdf

GIGA-D-18-00184_Revision_1.pdf

Response_to_Reviewer_Comments_Original_Submission.pdf

Reviewer_1_Report_(Original_Submission) -- Ekaterina Avershina 6/12/2018 Reviewed

Reviewer_2_Report_(Original_Submission) -- Zaid Abdo 6/15/2018 Reviewed

Supplemental Files
