# Supplementary material for: The metagenome of the female upper reproductive tract
Source: Gigascience. 2018 Sep 6;7(10):giy107. doi: 10.1093/gigascience/giy107 (PMC6177736; doi:10.1093/gigascience/giy107)
Supplement: GIGA-D-18-00184_Revision_1.pdf [file giy107_giga-d-18-00184_revision_1.pdf]

GigaScience

The metagenome of the female upper reproductive tract

--Manuscript Draft--

**Manuscript Number:**	GIGA-D-18-00184R1
**Full Title:**	The metagenome of the female upper reproductive tract
**Article Type:**	Research
**Funding Information:**	Shenzhen Municipal Government of China (JCYJ20160229172757249) — Mrs Chen Chen<br>Shenzhen Municipal Government of China (JCYJ20150601090833370) — Mrs Chen Chen<br>Macau Technology Development Fund (102/2016/A3) — Mrs Chen Chen
**Abstract:**	Background<br>The human uterus is traditionally believed to be sterile, while the vaginal microbiota plays important role in fending off pathogens. Emerging evidence demonstrates the presence of bacteria beyond the vagina. However, a microbiome-wide metagenomic analysis identifying the overall microorganism communities has been lacking.<br>Results<br>We performed shotgun-sequencing by Illumina platform of 52 samples from the cervical canal and the peritoneal fluid of Chinese women in reproductive age. Direct annotation of sequencing reads identified the taxonomy of bacteria, archaea, fungi and viruses, confirming and extending the results from our previous study. We replicated the findings in another 24 samples from the vagina, the cervical canal, the uterus and the peritoneal fluid using BGISEQ-500 platform, revealing that microorganisms in the samples from the same individual were largely shared in the whole reproductive tract. Over 99% human sequences were detected in the 20GB raw data. After filtering, vaginal microorganisms were well covered in the generated reproductive tract gene catalogue, while the more diverse upper reproductive tract microbiota might need greater depth of sequencing and more samples to meet the full coverage scale.<br>Conclusions<br>Microbiota in unprecedented data for unchartered body site, female upper reproductive tract, were analyzed in this study. The community results indicated that an intra-individual continuum of all types of microorganisms gradually changed from the vagina to the peritoneal fluid. A framework was also established in this study aiming at understanding the implications of the composition and functional potential of this distinct microbial ecosystem in relation to health and disease.
**Corresponding Author:**	Huijue Jia<br><br>CHINA
**Corresponding Author Secondary Information:**	
**Corresponding Author's Institution:**	
**Corresponding Author's Secondary Institution:**	
**First Author:**	Chen Chen
**First Author Secondary Information:**	
**Order of Authors:**	Chen Chen<br>Fei Li<br>Weixia Wei<br>Zirong Wang<br>Juanjuan Dai<br>Lilan Hao

Powered by Editorial Manager® and ProduXion Manager® from Aries Systems Corporation

	Liju Song
	Xiaowei Zhang
	Liping Zeng
	Hui Du
	Huiru Tang
	Na Liu
	Huanming Yang
	Jian Wang
	Lise Madsen
	Susanne Brix
	Karsten Kristiansen
	Xun Xu
	Junhua Li
	Ruifang Wu
	Huijue Jia
**Order of Authors Secondary Information:**	
**Response to Reviewers:**	Reviewer reports:<br>Reviewer #1: The manuscript has vastly improved since the first submission, although some additional information and language editing is required.<br><br>I would like to thank the authors for including more in-depth information about the functional data; i think p-values for the aforementioned differences in gene families have to be provided; maybe marking the significant differences on the figure. I also think that since this is actually the most novel data in the paper, the Supplementary Figure 3 should be moved to the main body of the paper.<br>We thank reviewer for the valuable comment. For the functional data, we evaluated the difference between the CV and PF by comparing all the functional genes from the built catalogues instead of the individual samples to avoid the deviation. Hence, we can’t do the statistical analysis, such as p-value calculation. Additionally, we agreed with the suggestion from reviewer and moved Supplementary Figure 3 to the main body to make the paper more fulness and integrated.<br><br>Authors have enough of samples to claim the gradient in microbiota over the reproductory tract; as well as enough of samples to perform functional comparisons between PF and CU samples, but believe that these data is not enough to address differences in alpha- and beta-diversity between PF/CU. I find this argument a little vague and I strongly believe that the paper would benefit from including this information, but since it initially was just my suggestion to the authors, they are free to ignore it.<br>We would like to thank the reviewer’s question. We consider that the sample number of PF is enough to explain the microbiota and the community function since the microbiota and its function were mainly dressed by the dominate species which could by fully explained by the data achieved from these samples. However, we claim that it is not enough to address differences in alpha- and beta-diversity between PF/CU because that the rarefaction curve of PF did not reach the saturation, while CU did. That means some species may still not been detected in the PF samples and this will cause the deviation of the bacterial alpha- and beta-diversity results.<br><br>Line 161, figure 3, the PF line is not far from reaching the plateu - it does seem that the line is approaching the asymptote, so i belive that 'far from saturation' is an overstatement and should be toned down.<br>We agree with the suggestion raised from the reviewer. After the double checking, we totally agreed with the reviewer and revised this statement to make it toned down (Line 160-162).

	Reviewer #2: Still think that a quick read for English would be useful for this manuscript though the writing has improved. Examples of writing that needs correcting are as follows:<br>Lines 34-35 in the abstract: "the vaginal microbiota plays important roles … " should be "role"; Line 91 "(the stringent selection rules …" should be "(for the stringent selection rules …"<br>We thank reviewer very much for the carefully reading and correction. We revised the manuscript according to the comments and checked the English carefully throughout the manuscript.<br><br>The clustering process is still not described. Did the authors use hierarchical clustering, kmeans clustering …. What was the cutoff used to identify the clusters? Based on this and on figure Sup Fig 2 I don't believe that they achieved individual sub-cluster representation.<br>We thank reviewer for pointing out this information which should not be omitted. We applied centroid-linkage method for the hierarchical clustering in this study and the detailed information has been added in the methods (Line 194-195). After hierarchical clustering, we selected the samples with enough DNA amount in each sub-cluster as the representative candidates to do the further analysis.<br><br>Line 92 Supplementary Figure 2 does not provide details of the stringent selection rules.<br>We would like to thank the reviewer for the question. The reason why we cite Supplementary Fig. 2 here is one of the selection rules is according to the results of the clustering which presented in the Supplementary Fig. 2. However, as the reviewer pointed out, this figure did not provide the details of the stringent selection rules, so we deleted the citation in line 92.<br><br>Line 136 The authors should provide the reference to the "previous" study when they mention it.<br>We are grateful for the reviewer's suggestion. We agreed with the reviewer and added the reference here, which is our recent study using 16S rRNA amplicon sequencing.
**Additional Information:**	
**Question**	**Response**
Are you submitting this manuscript to a special series or article collection?	No
**Experimental design and statistics**<br><br>Full details of the experimental design and statistical methods used should be given in the Methods section, as detailed in our Minimum Standards Reporting Checklist. Information essential to interpreting the data presented should be made available in the figure legends.<br><br>Have you included all the information requested in your manuscript?	Yes
**Resources**	Yes

Powered by Editorial Manager® and ProduXion Manager® from Aries Systems Corporation

A description of all resources used, including antibodies, cell lines, animals and software tools, with enough information to allow them to be uniquely identified, should be included in the Methods section. Authors are strongly encouraged to cite Research Resource Identifiers (RRIDs) for antibodies, model organisms and tools, where possible.<br><br>Have you included the information requested as detailed in our Minimum Standards Reporting Checklist?	
**Availability of data and materials**<br><br>All datasets and code on which the conclusions of the paper rely must be either included in your submission or deposited in publicly available repositories (where available and ethically appropriate), referencing such data using a unique identifier in the references and in the “Availability of Data and Materials” section of your manuscript.<br><br>Have you have met the above requirement as detailed in our Minimum Standards Reporting Checklist?	Yes

Powered by Editorial Manager® and ProduXion Manager® from Aries Systems Corporation

Manuscript

Click here to access/download;Manuscript;manuscript_20180627_SCE.doc

Click here to view linked References

The metagenome of the female upper reproductive tract

Chen Chen[2,3,*], Fei Li[1,2,3*], Weixia Wei[4,5,*], Zirong Wang[2,], Juanjuan Dai[4,5,], Lilan Hao[2,3], Liju Song[2,3], Xiaowei Zhang[2,3], Liping Zeng[4,5], Hui Du[4,5], Huiru Tang[4,5], Na Liu[6], Huanming Yang[2,9], Jian Wang[2,9], Lise Madsen[2,7,11], Susanne Brix[12], Karsten Kristiansen[2,7], Xun Xu[2,3], Junhua Li[2,3,8,13,], Ruifang Wu[4,5†], Huijue Jia[2,3,8,10,†]

[1]BGI Education Center, University of Chinese Academy of Sciences, Shenzhen 518083, China.

[2]BGI-Shenzhen, Shenzhen 518083, China.

[3]China National GeneBank, BGI-Shenzhen, Shenzhen 518120, China.

[4]Peking University Shenzhen Hospital, Shenzhen 518036, China.

[5]Shenzhen Key Laboratory on Technology for Early Diagnosis of Major Gynecological diseases, Shenzhen, PR China

[6]BGI genomics, BGI-Shenzhen, Shenzhen 518083, China

[7]Laboratory of Genomics and Molecular Biomedicine, Department of Biology, University of Copenhagen, Universitetsparken 13, 2100 Copenhagen, Denmark.

[8]Shenzhen Key Laboratory of Human Commensal Microorganisms and Health Research, BGI-Shenzhen, Shenzhen 518083, China.

[9]James D. Watson Institute of Genome Sciences, Hangzhou310000, China.

[10]Macau University of Science and Technology, Taipa, Macau 999078, China.

[11]Institute of Marine Research (IMR), Postboks 1870, Nordnes, N-5817, Bergen, Norway.

[12]Department of Biotechnology and Biomedicine, Technical University of Denmark, Soltofts Plads, 2800 Kongens. Lyngby, Denmark.

[13]School of Bioscience and Biotechnology, South China University of Technology, Guangzhou 510006, China;

[*]These authors contributed equally to this work.

[†]Correspondence should be addressed to H. J. (jiahuijue@genomics.cn, ORCID: 0000-0002-3592-126X) or R. W. (wurf100@126.com).

Abstract

Background

The human uterus is traditionally believed to be sterile, while the vaginal microbiota plays important role in fending off pathogens. Emerging evidence demonstrates the presence of bacteria beyond the vagina. However, a microbiome-wide metagenomic analysis identifying the overall microorganism communities has been lacking.

Results

We performed shotgun-sequencing using the Illumina platform of 52 samples from the cervical canal and peritoneal fluid of Chinese women in reproductive age. Direct annotation of sequencing reads identified the taxonomy of bacteria, archaea, fungi and viruses, confirming and extending the results from our previous study. We replicated the findings in another 24 samples from the vagina, the cervical canal, the uterus and peritoneal fluid using BGISEQ-500 platform, revealing that microorganisms in the samples from the same individual were largely shared in the whole reproductive tract. Over 99% human sequences were detected in the 20GB raw data. After filtering, vaginal microorganisms were well covered in the generated reproductive tract gene catalogue, while the more diverse upper reproductive tract microbiota might need greater depth of sequencing and more samples to meet the full coverage scale.

Conclusions

Microbiota in unprecedented data for unchartered body site, female upper reproductive tract, were analyzed in this study. The community results indicated that an intra-individual continuum of all types of microorganisms gradually changed from the vagina to the peritoneal

fluid. A framework was also established in this study aiming at understanding the implications of the composition and functional potential of this distinct microbial ecosystem in relation to health and disease.

Keywords

Metagenomics, Microbiota, Female upper reproductive tract

Background

As humans evolved, the female reproductive tract has formed complex and unique structures such as the uterus, cervix and the vagina. The human vagina hosts trillions of bacteria that can significantly impact the health of women and their neonates. The cervix has been regarded to be a perfect barrier between the vagina and uterus leading to the assumption that the upper reproductive tract functions in a sterile environment. However, judging from evidence in insects and other animals, humans are probably no exception with regard to vertical transmission of the mothers' microbiota before birth [1]. Thus, in humans, bacterial DNA has been detected in the placenta [2,3]. Based on our recent analyses using 16S rRNA amplicon sequencing, the upper reproductive tract, including cervix, uterus, fallopian tubes and peritoneal fluid harbor diverse communities of bacteria, though at low abundance [4].

Recently, the studies of female reproductive tract microbiota have mainly focused on the vagina using 16S rRNA amplicon sequencing [5–7]. Studies using 16S rRNA gene amplicon sequencing have limitations in relation to lower taxonomic resolution and the lack of ability to perform species-specific functional inference. Metagenomic shotgun sequencing can address these limitations, but only a few studies have applied metagenomic shotgun sequencing on the

vaginal microbiota [8], and no studies have characterized the compositional range of the upper reproductive tract microbiome using metagenomic analysis. The present study is the first to provide metagenomic data from the female upper reproductive tract.

Data description

Samples of six locations (CL, lower third of vagina; CU, posterior fornix; CV, cervical mucus drawn from the cervical canal; ET, endometrium; FLL and FRL, left and right fallopian tubes; PF, peritoneal fluid from the pouch of Douglas) throughout the female reproductive tract from 137 Chinese women of reproductive age, undergoing surgery for conditions not known to involve infection (**Supplementary Table 1**) were collected for this study. 16S rRNA gene amplicon sequencing was performed on 665 of these samples. The results from 476 of these have been published previously [4], and those from the remaining 189 were presented in this study. Two samples (1 CV and 1 CU) were subjected to shotgun sequencing with or without prior removal of human DNA using a commercial kit to test the experimental effect of host sequencing removing (refer to **Methods** section). Then, 25 PF and 25 CV samples were sequenced on the Illumina HiSeq platform using 100 bp paired-end (PE) sequencing (for the stringent selection rules of samples, see **Methods** for details). For these 52 samples, 20GB of raw data per sample, corresponding to a total of 0.99 TB were generated. Additionally, intra-individual similarity in the vagino-uterine microbiota were also examined basing on 24 samples from different sites of the reproductive tract (CL, CU, CV, ET, PF) in 6 women. These samples were sequenced on the BGISEQ-500 sequencer using 100 bp single-end (SE) sequencing and generated 60GB of raw data per sample, totaling 1.40 TB. The dataset after

filtering out low-quality and host reads (refer to **Methods** section) is available via the EBI database using the accession number PRJEB24147.

Analyses and Discussion

Metagenomic sequencing

According to shotgun-sequencing of vaginal samples by the Human Microbiome Project (HMP) and of placental samples by Aagaard *et al.*, over 90% of the sequences were derived from human host DNA [2,9]. To overcome this problem, we first tested a commercial kit that removes human DNA by binding and precipitating CpG-methylated DNA. Unfortunately, after the kit treatment, a considerable amount (99.9% for CV sample and 79% for CU sample) of host DNA still remained (**Supplementary Fig. 1a**). Furthermore, the bacteria compositions varied by kit treatment when comparing with the control group (**Supplementary Fig. 1b**). We therefore abandoned the strategy of host DNA removal prior to shotgun metagenomics sequencing.

The sample selection was founded on the data from CV and PF samples [4], which we identified as robust representations of the overall samples. Since higher amounts of DNA is required for shotgun-sequencing results, a more stringent rule was set as the following two criteria: individual sub-clusters representation and sufficient DNA amount (see details in **Methods** section). To follow the former criterion, clustering results based on the relative abundances of OTUs in the PF and CV samples showed that the samples marked with red (all containing DNA > 1 μg) were well distributed amongst all collected samples (**Supplementary Fig. 2**), so these were selected for shotgun-sequencing in this study. As a

result, 25 PF and 25 CV samples were selected for sequencing using the Illumina HiSeq 4000 platform. After quality control, high-quality reads were aligned to hg 19 using SOAP and GRCh38 using DeconSeq to remove human reads (see details in **Methods** section). The average host contamination rate of 99.72% for CV and 99.93% for PF (**Supplementary Table 2**), which were lower than that previously reported for placenta samples [2].

The findings further expanded by inclusion of additional 24 samples subjected to sequencing on the BGISEQ-500 platform, in which we also examined the intra-individual similarity in the vagino-uterine microbiota based on samples from different sites of the reproductive tract (CL, CU, CV, ET, PF). The average host contamination rate for vagina (CL, CU) samples was 96.55%, and lower than those of the CV, ET and PF samples, which all above 99.5% (**Supplementary Table 2**).

A diverse microbiome in the cervical canal and the peritoneal fluid of reproductive age women

To obtain an overview of the overall composition of the vagino-uterine microbiome, we used Kraken to directly assign sequencing reads to all types of microbial taxa [10]. The dominant *Lactobacillus* spp. in CV and *Pseudomonas* spp. in PF were detected in the present study and in corresponded with the previous study [4]. In addition, the microbiome that comprise methane-producing archaea, yeasts, herpesviruses, papillomaviruses, and bacteriophages were also founded (**Fig. 1a, b**).

The abundance of these taxonomic units varied among samples, and those constituting more than 0.1% of the total reads number were identified in the CV and PF samples from the same individual (**Fig. 1c**).

To gain further insight into compositional similarities of the microbiota at different sites of the reproductive tract in the same individual, we selected taxa at the family level which fulfilled two criteria: they were presented in at least two sites of the same individual and the relative abundance was higher than 0.1%. Taxa fulfilling these criteria made up more than 45% of the microorganisms presented in the samples across the 6 individuals subjected for this detailed analysis (**Fig. 2**). Lactobacillaceae or Bifidobacteriaceae dominated in vagina (CL and CU), but not in the upper reproductive tract, where microorganisms such as Pseudomonadaceae, Propionibacteriaceae, Streptococcaceae and Moraxellaceae constituted a notable fraction of the microbiota. In addition, eukaryotes, viruses and archaea, such as Saccharomycetaceae, Herpesviridae, Ferroplasmaceae were also found in the female reproductive tract. The results at the bacterial level are in keeping with our findings in a recent study [4], and the current data further demonstrates an intra-individual continuum of all types of microorganisms that gradually changes from the vagina to the peritoneal fluid.

Genes from the vagino-uterine microbiota

Reference gene catalogs have greatly facilitated analyses of the microbiome, especially the human gut microbiome [11–13]. Here, we established the first gene catalog of the microbiome of the female upper reproductive tract, which comprises of 60,699 genes. Rarefaction analysis based on gene number revealed a curve approaching saturation with about 23 CV samples (**Fig. 3**). However, rarefaction analysis based on gene numbers in PF samples revealed a curve that close to saturation but still did not reach the plateau, possibly due to a more diverse microbiota in PF. Therefore, with 20GB sequences per sample, vaginal bacteria could be well covered, whereas characterization of bacteria from the upper

reproductive tract would require a higher amount of sequences and more samples.

We annotated the genes in the gene catalog according to the Kyoto Encyclopedia of Genes and Genomes (KEGG) [14]. The matched genes in PF (15,316 genes) were all covered within CV (39,087 genes). Comparing CV and PF in the distribution of KEGG pathways, PF showed a greater proportion of genes in carbohydrate metabolism, replication and repair, membrane transport and drug resistance, whereas the genes involved in translation, energy metabolism and metabolism of cofactors and vitamins were enriched in CV (**Fig. 4**). In KO modules, CV showed enrichment of transport systems for thiamine, cystine, teichoic acid, taurine and putative ABC transport systems compared to PF. Regulatory systems of aerobic and anaerobic respiration, osmotic stress response and multicellular behavior control also enriched in CV (**Supplementary Table 3**).

Methods

Sample description

A total of 137 Chinese women of reproductive age, undergoing surgery for conditions not known to involve infection (hysteromyoma, adenomyosis, endometriosis, and salpingemphraxis) were enrolled in this study (**Supplementary Table 1**). Samples were taken from the CL, CU and CV on the day of the clinical visit without any prior disturbance. Depending on the clinical conditions, laparoscopy or laparotomy were performed, and samples from the ET, FLL, FRL and PF were taken during surgery (**Supplementary Table 1**). The study was approved by the institutional review boards at Peking University Shenzhen Hospital and BGI-Shenzhen, and all women provided written informed consent. The subject

exclusion criteria, sampling and DNA extraction methods can be found in [4].

To test the effect of experimental removal of human DNA, one CU sample and one CV sample were used to shotgun sequencing on Illumina HiSeq2000 platform with or without prior removal of human DNA, respectively. The NEBNext Microbiome DNA Enrichment Kit was used here according to the manufacturer's instructions with a total of 10 μg input DNA per sample.

Then we made a prior selection of samples to undergo shotgun-sequencing. The selection was founded on the data from CV and PF samples [4] based on the following two criteria: i) samples should represent individual sub-clusters when subjected to hierarchical (centroid-linkage) clustering based on relative abundances of operational taxonomic units (OTUs) from 16S rRNA gene amplicon sequencing; ii) the amount of DNA should be above 1 μg. The samples with good scattering in different clusters based on the relative abundances of OTUs in the PF and CV samples were selected for shotgun-sequencing on Illumina HiSeq4000 platform.

We replicated the findings in another 24 samples on the BGISEQ-500 platform, where additional sites (CL, CU, CV, ET and PF) of 6 women were moreover involved. To meet the need of library construction, the amount of DNA in the all 24 samples were above 1 μg. And three qualified samples for each woman were set as a threshold.

Metagenomic shotgun sequencing

Library construction and shotgun sequencing using Illumina HiSeq2000/4000 platforms (insert size 350 bp; 100 bp of PE reads; two replicate libraries were constructed for each lane.)

and BGISEQ-500 (100 bp of SE reads; one library was constructed for each lane) were performed as previously described [15] (and see protocol in protocols.io[16]). The quality control of sequencing data from the HiSeq and BGISEQ platforms were also followed this study. Then, human sequences were eliminated by alignment to the hg19 reference genome using SOAP2.22 (SOAPaligner/soap2, RRID:SCR_005503). As the resulting data still contained human sequences, a more stringent procedure using DeconSeq by aligning data to the GRCh38 reference genome was applied [17].

Taxonomic assignment of sequencing reads

High-quality, non-human sequences were tentatively assigned to microbial taxa using Kraken with default parameters (Kraken, RRID:SCR_005484)[10]. For pair-end reads Kraken concatenated the pairs together with a single N between the sequences automatically with default parameters and the manual clarified that this software raised the sensitivity by about 3 percentage points over classifying the sequences as single-end reads.

Construction of a gene catalog

The high-quality, non-human sequencing reads of 52 samples sequenced by Illumina HiSeq platforms were *de novo* assembled into contigs using IDBA-UD (IDBA-UD (RRID:SCR_011912))[18]. We used the same strategy as previous study [12,13], where genes were predicted from the contigs by MetaGeneMark [19], and highly similar genes (95% identity, 90% overlap) were removed as redundancy using CD-HIT (CD-HIT, RRID:SCR_007105) [20]. Functional annotations were made by BLASTP (v2.2.24) based on KEGG (v76) databases (KEGG , RRID:SCR_012773)[14].

Availability of Supporting Data

The sequencing data after filtering out low-quality and host reads is available via the EBI database using the accession number PRJEB24147. Additional supporting data is available via the *GigaScience* GigaDB database [21].

Abbreviations

bp: base pair; GB: Gigabase; HMP: Human Microbiome Project; KEGG: Kyoto Encyclopedia of Genes and Genomes; OTU: operational taxonomic units; PE: paired-end; SE: single-end.

Author's contributions

H.J. and R.W. conceived and directed the project. W.W., J.D., L.Z., H.D., H.T., and R.W. performed the clinical diagnosis, sample collection. C.C., Z.W., F.L., and L.H. performed the bioinformatic analyses and prepared display items. C.C., F.L., Z.W., X.Z., J.L. and H.J. wrote the first version of the manuscript. L.M., S.B. and K.K. revised the manuscript. All authors contributed to the final revision of the manuscript.

Acknowledgements

The study was supported by the Shenzhen Municipal Government of China (JCYJ20160229172757249, JCYJ20150601090833370), the grant from the Macau Technology Development Fund (102/2016/A3). We gratefully acknowledge colleagues at BGI-Shenzhen for DNA quality control, library construction, sequencing, and helpful discussions.

Competing financial interests

The authors declare no competing financial interests.

References

1. Funkhouser LJ, Bordenstein SR. Mom Knows Best: The Universality of Maternal Microbial Transmission. PLoS Biol. 2013;11:e1001631.

2. Aagaard K, Ma J, Antony KM, Ganu R, Petrosino J, Versalovic J. The placenta harbors a unique microbiome. Sci. Transl. Med. 2014;6:237ra65.

3. Antony KM, Ma J, Mitchell KB, Racusin DA, Versalovic J, Aagaard K. The Preterm Placental Microbiome Varies in Association with Excess Maternal Gestational Weight Gain. Am J Obs. Gynecol. Elsevier; 2014;212:653.e1-653.e16.

4. Chen C, Song X, Wei W, Zhong H, Dai J, Lan Z, et al. The microbiota continuum along the female reproductive tract and its relation to uterine-related diseases. Nat Commun. 2017 Oct 17;8(1):875. doi: 10.1038/s41467-017-00901-0.

5. Ravel J, Gajer P, Abdo Z, Schneider GM, Koenig SSK, Mcculle SL, et al. Vaginal microbiome of reproductive-age women. Proc. Natl. Acad. Sci. 2010;108:4680–7.

6. Gajer P, Brotman RM, Bai G, Sakamoto J, Schutte UME, Zhong X, et al. Temporal Dynamics of the Human Vaginal Microbiota. Sci. Transl. Med. 2012;4:132ra52-132ra52.

7. Ding T, Schloss PD. Dynamics and associations of microbial community types across the human body. Nature. 2014 May 15;509(7500):357-60. doi: 10.1038/nature13178.

8. Lloyd-price J, Mahurkar A, Rahnavard G, Crabtree J, Orvis J, Hall AB, et al. Strains , functions and dynamics in the expanded Human Microbiome Project. Nature. 2017 Oct 5;550(7674):61-66. doi: 10.1038/nature23889.

9. Methé BA, Nelson KE, Pop M, Creasy HH, Giglio MG, Huttenhower C, et al. A framework for human microbiome research. Nature. 2012;486:215–21.

10. Wood DE, Salzberg SL. Kraken: ultrafast metagenomic sequence classification using exact alignments. Genome Biol. 2014;15:R46.

11. Wang J, Jia H. Metagenome-wide association studies: fine-mining the microbiome. Nat. Rev. Microbiol. 2016;14:508–22.

12. Li J, Jia H, Cai X, Zhong H, Feng Q, Sunagawa S, et al. An integrated catalog of reference genes in the human gut microbiome. Nat. Biotechnol. 2014;32:834–41.

13. Xie H, Guo R, Zhong H, Feng Q, Lan Z, Qin B, et al. Shotgun Metagenomics of 250 Adult Twins Reveals Genetic and Environmental Impacts on the Gut Microbiome. Cell Syst. 2016;

14. Kanehisa M, Sato Y, Kawashima M, Furumichi M, Tanabe M. KEGG as a reference resource for gene and protein annotation. 2016;44:457–62.

15. Fang C, Zhong H, Lin Y, Chen B, Han M, Ren H, et al. Assessment of the cPAS-based BGISEQ-500 platform for metagenomic sequencing. Gigascience. 2018 Mar 1;7(3):1-8. doi: 10.1093/gigascience/gix133.

16. Jie Huang, Xinming Liang, Yuankai Xuan, Chunyu Geng, Yuxiang Li, Haorong Lu, Shoufang Qu, Xianglin Mei, Hongbo Chen, Ting Yu, Nan Sun, Junhua Rao, Jiahao Wang, Wenwei Zhang, Ying Chen, Sha Liao, Hui Jiang, Xin Liu, Zhaopeng Yang, Feng Mu, Shangxian Gao (2018). BGISEQ-500 WGS library construction. protocols.io dx.doi.org/10.17504/protocols.io.ps5dng6

17. Schmieder R, Edwards R. Fast identification and removal of sequence contamination from genomic and metagenomic datasets. PLoS One. 2011;6.

18. Peng Y, Leung HCM, Yiu SM, Chin FYL. IDBA-UD: a de novo assembler for single-cell and metagenomic sequencing data with highly uneven depth. Bioinformatics. 2012;28:1420–8.

19. Hyatt D, Locascio PF, Hauser LJ, Uberbacher EC. Gene and translation initiation site prediction in metagenomic sequences. Bioinformatics. 2012;28:2223–30.

20. Li W, Godzik A. Cd-hit: a fast program for clustering and comparing large sets of protein or nucleotide sequences. Bioinformatics. 2006;22:1658–9.

21. Chen C; Li F; Wei W; Wang Z; Dai J; Hao L; Song L; Zhang X; Zeng L; Du H; Tang H; Liu N; Yang H; Wang J; Madsen L; Brix S; Kristiansen K; Xu X; Li J; Wu R; Jia H (2018): Supporting data for "The metagenome of the female upper reproductive tract" GigaScience Database. http://dx.doi.org/10.5524/100491

Figure legends

Figure 1: The overall microbiome composition of the cervical canal and the peritoneal fluid of reproductive age women. Cumulative bar charts of the main taxa at domain (**a**) and family (**b**) levels in CV and PF samples. (**c**) Compositional overlap at family level of CV and PF samples from the same individuals. Relative number of reads was calculated as $N_p = \frac{a_p}{a_t} \times m$, where a_p is the number of reads within p taxa in a sample. a_t is the total number of reads within a sample, m is median number of reads within all 50 samples. When p taxa is shared by CV and PF samples from the same individuals, and at the same time both N_p values are higher than $0.1\% \times m$, the p taxa is included in the cumulative bar charts. Taxa names (**b**, **c**) in black, purple, and blue denote bacteria, eukaryote and viruses, respectively.

Figure 2: Composition of the vagino-uterine microbiota. (**a**, **c**, **e**, **g**, **i**, **k**) Venn diagram depicting shared taxa at the family levels in samples collected at different sites in the same individual. (**b**, **d**, **f**, **h**, **k**, **l**) Cumulative bar charts of the taxa with relative abundance higher than 0.1% and present in at least two sites of the same individual. Taxa names (**b**, **c**) in black, purple, blue, and grey denote bacteria, eukaryote, viruses and archaea, respectively.

Figure 3: Rarefaction of microbial gene content in CV (a) and PF (b) samples. The number of genes in each group was calculated after 100 random samplings with replacement. Boxes denote the interquartile range (IQR) between the first and third quartiles (25th and 75th percentiles, respectively) and the line inside denotes the median. Whiskers denote the lowest and highest values within 1.5 times IQR from the first and third quartiles, respectively.

Circles denote outliers beyond the whiskers.

Figure 4: KEGG pathway classification of the vagino-uterine microbiome. Comparison of CV (red) and PF (blue) data based on KEGG annotation, which emphasizes functional similarity of the CV and PF microbiota.

Supplementary Figure legends

Supplementary Figure 1: Evaluation of the NEBNext Microbiome DNA Enrichment Kit by two comparative strategies. Sample names suffixed by "-HR" represent DNA samples that were treated with the kit for removal of host DNA before shotgun sequencing, while sample names suffixed by A represent DNA samples that were subjected to shotgun sequencing directly (**a**). The table data shows the obtained read number, and remaining reads after removal of host DNA reads in the two samples. **b**) Influence of host DNA presence on bacterial DNA identification during shotgun sequencing. The plots display the compositional difference amongst major bacteria genera in samples with and without (-HR) host DNA presence. Data were analyzed by mapping reads to the ICG bacterial reference gene catalog [12].

Supplementary Figure 2: Samples selected for metagenomic sequencing. Hierarchical clustering of CV (**a**) and PF (**b**) samples based on the relative abundances of OTUs. Samples which represent individual sub-clusters and hold DNA amounts above 1 μg were selected for shotgun-sequencing (red).

Figure 1

Click here to access/download;Figure;FIG1-20180126.pdf

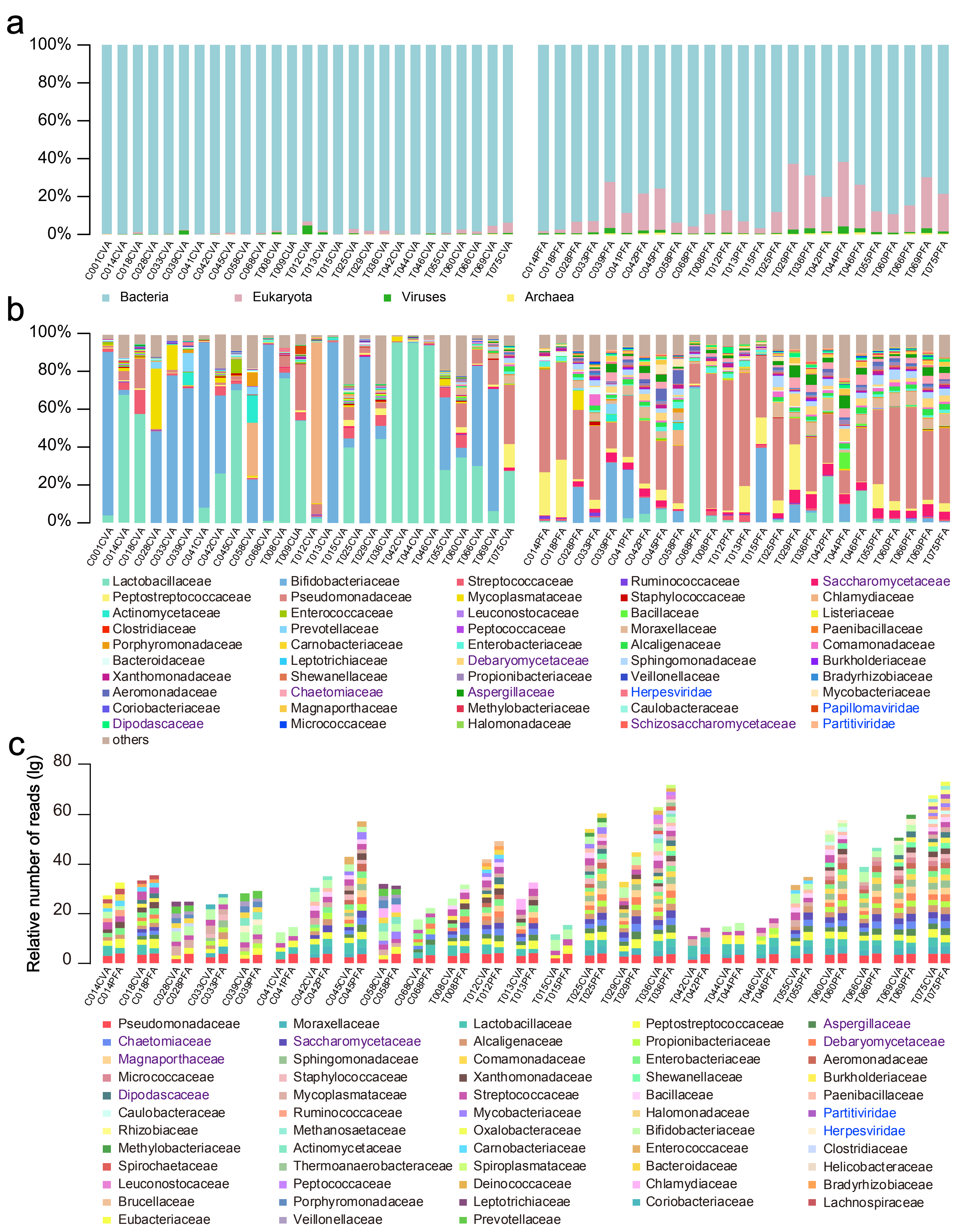

Figure2
Click here to access/download;Figure;FIG2-20180126.pdf

Figure 2

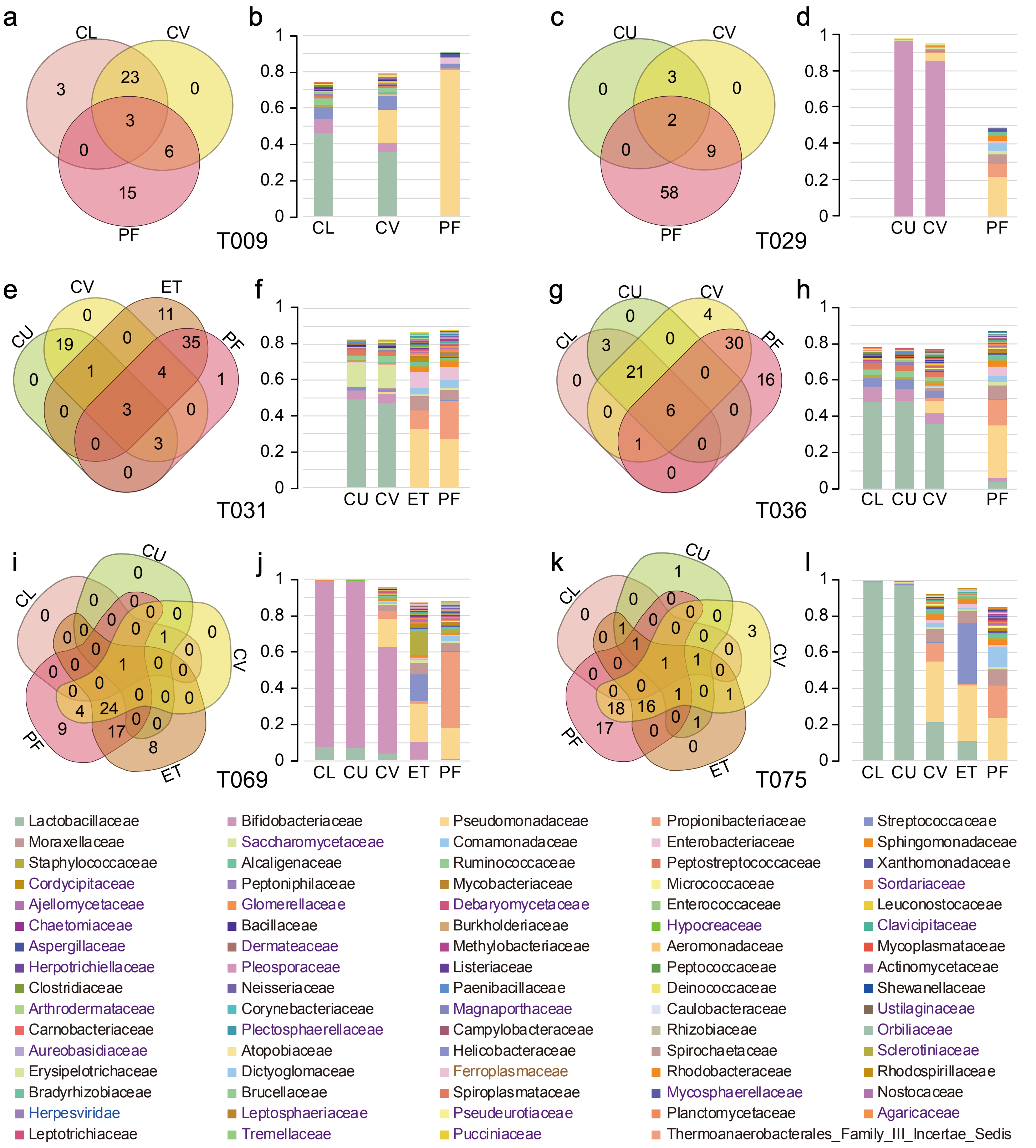

Figure3 Click here to access/download;Figure;FIG3-20180126.pdf

Figure 3

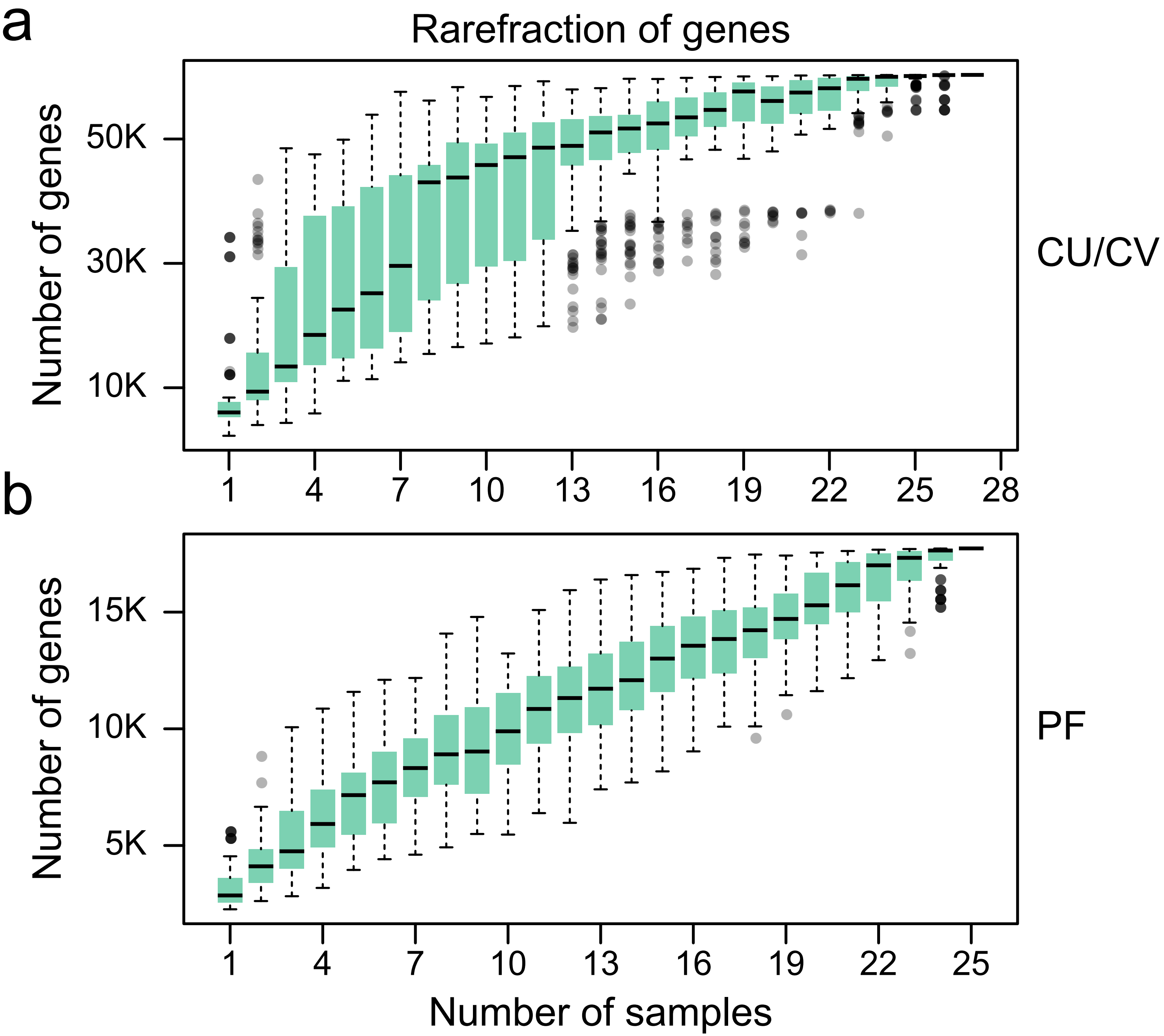

Click here to access/download;Figure;FIG4.pdf

Figure 4

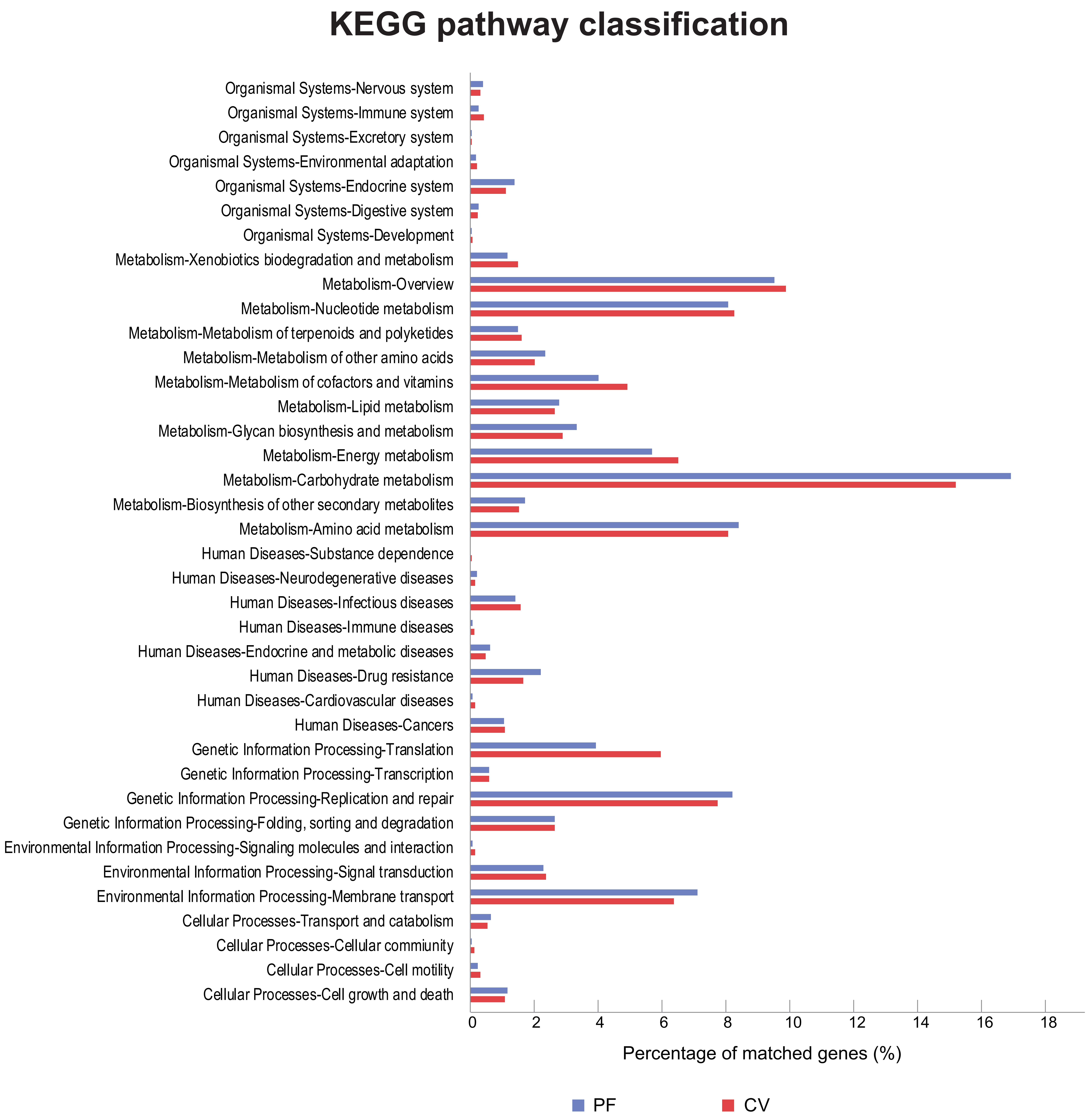

Supplementary Figure 1

Click here to access/download
Supplementary Material
SFIG1-20180126.pdf

Supplementary Figure 2

Click here to access/download
Supplementary Material
SFIG2-20180126.pdf

Supplementary Table1

Click here to access/download
Supplementary Material
Supplementary Table 1-20180126.xlsx

Supplementary Table2

Click here to access/download
Supplementary Material
Supplementary Table 2-20180126.xlsx

Supplementary Table3

Click here to access/download
Supplementary Material
Supplementary Table 3.xlsx
