# Supplementary material for: The metagenome of the female upper reproductive tract
Source: Gigascience. 2018 Sep 6;7(10):giy107. doi: 10.1093/gigascience/giy107 (PMC6177736; doi:10.1093/gigascience/giy107)

Supplementary Figure 1

a

| Sample name | # Raw reads | # High quality reads | # Reads after host removal | % Host reads |
|---|---|---|---|---|
| C001CVA | 243,578,598 | 231,546,980 | 130,126 | 99.94 |
| C001CV-HR | 59,353,258 | 56,228,220 | 55,938 | 99.90 |
| T009CUA | 241,994,266 | 229,816,118 | 5,225,740 | 97.73 |
| T009CU-HR | 51,937,996 | 49,020,066 | 10,294,854 | 79.00 |

b

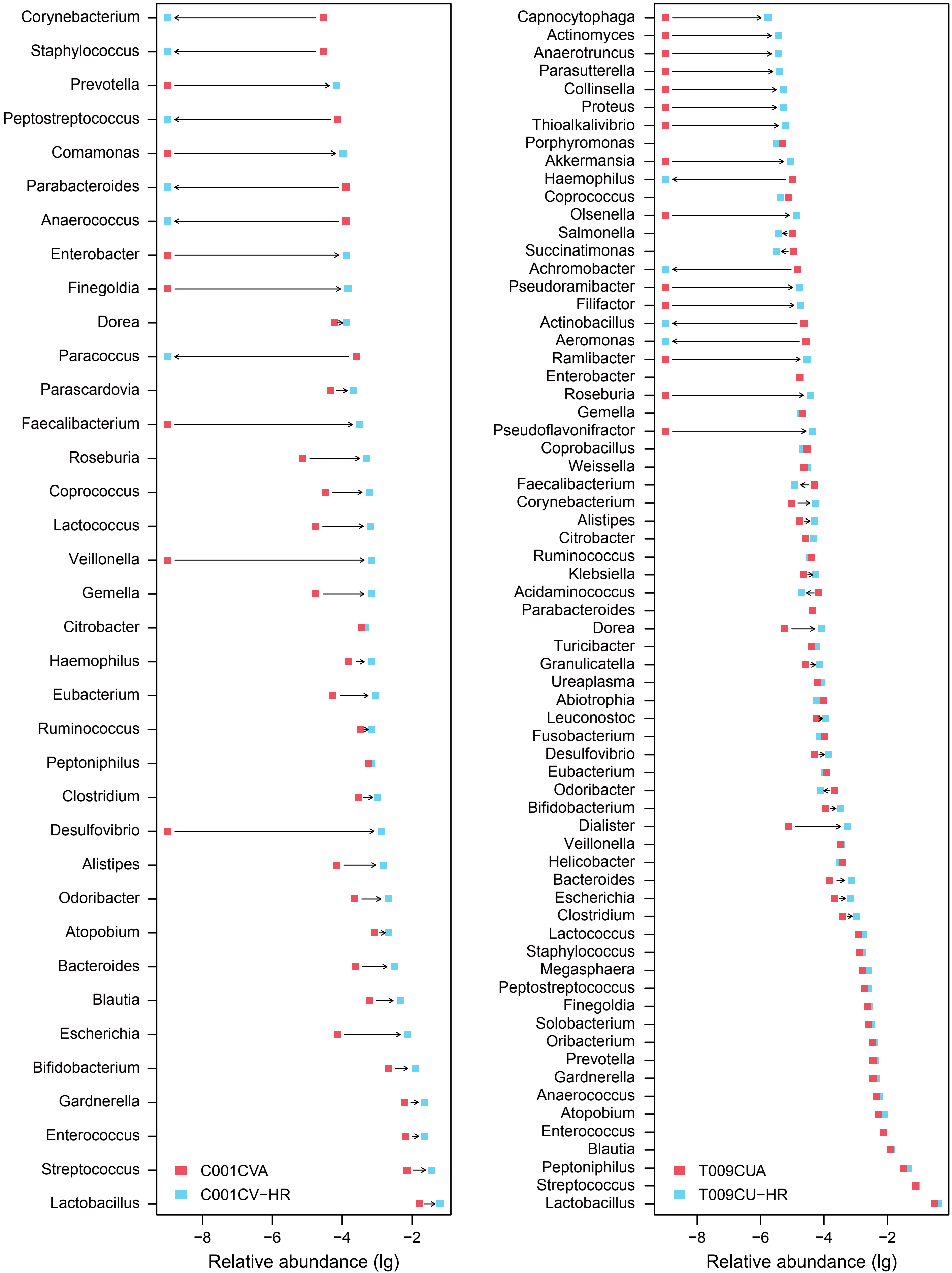

Supplement: Supplemental Files [file giy107_supplemental_files.zip › SFIG1-20180126.pdf]
