# Supplementary figures and images for: The metagenome of the female upper reproductive tract
Source: Gigascience. 2018 Sep 6;7(10):giy107. doi: 10.1093/gigascience/giy107 (PMC6177736; doi:10.1093/gigascience/giy107)

Supplementary Figure 2

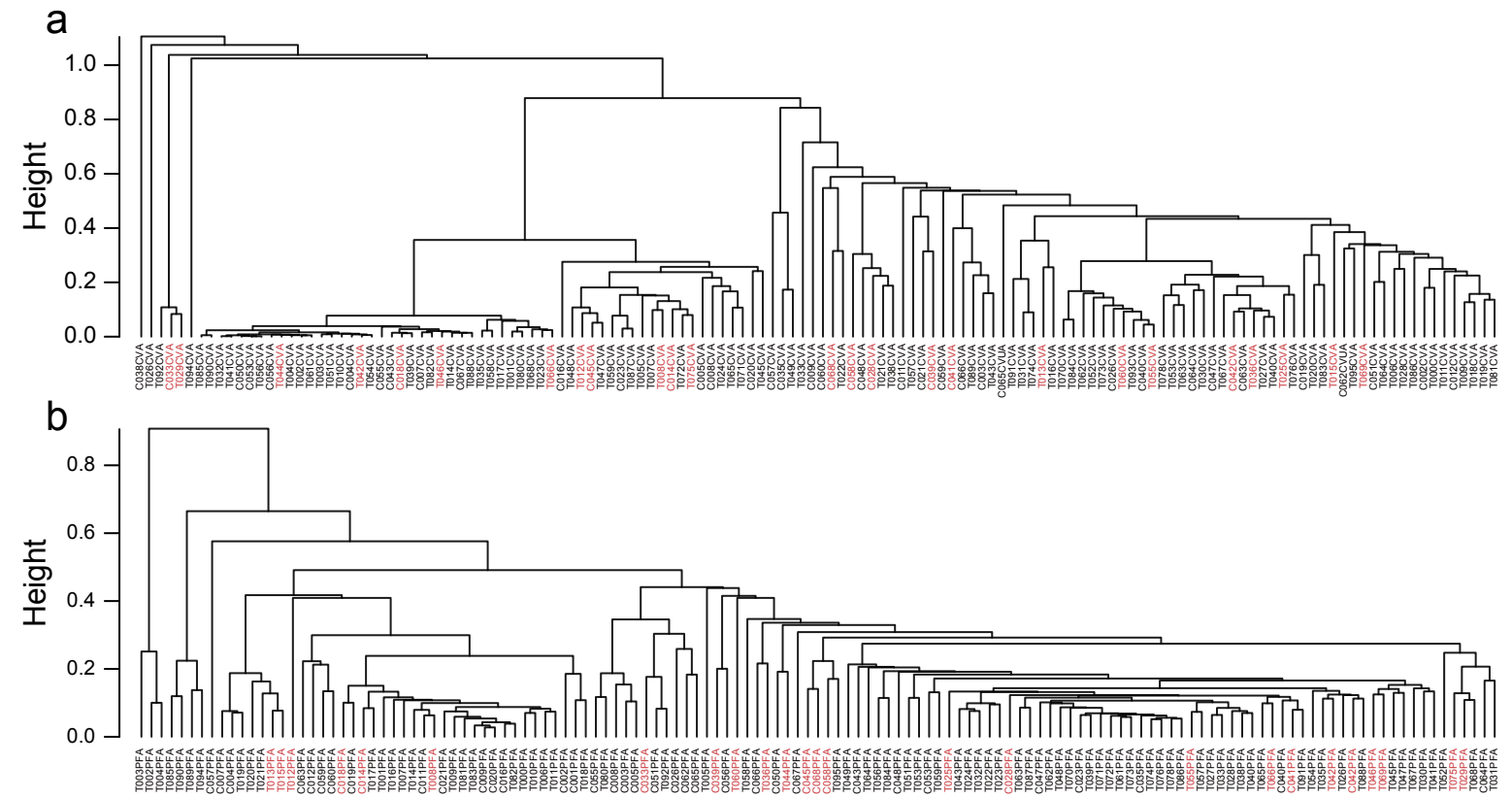

Supplement: Supplemental Files [file giy107_supplemental_files.zip › SFIG2-20180126.pdf]
