# Supplementary material for: The metagenome of the female upper reproductive tract
Source: Gigascience. 2018 Sep 6;7(10):giy107. doi: 10.1093/gigascience/giy107 (PMC6177736; doi:10.1093/gigascience/giy107)
Supplement: Response_to_Reviewer_Comments_Original_Submission.pdf [file giy107_response_to_reviewer_comments_original_submission.pdf]

Author's Response To Reviewer Comments

Close

Reviewer reports:
Reviewer #1: The manuscript has vastly improved since the first submission, although some additional information and language editing is required.

I would like to thank the authors for including more in-depth information about the functional data; i think p-values for the aforementioned differences in gene families have to be provided; maybe marking the significant differences on the figure. I also think that since this is actually the most novel data in the paper, the Supplementary Figure 3 should be moved to the main body of the paper.
We thank reviewer for the valuable comment. For the functional data, we evaluated the difference between the CV and PF by comparing all the functional genes from the built catalogues instead of the individual samples to avoid the deviation. Hence, we can't do the statistical analysis, such as p-value calculation. Additionally, we agreed with the suggestion from reviewer and moved Supplementary Figure 3 to the main body to make the paper more fulness and integrated.

Authors have enough of samples to claim the gradient in microbiota over the reproductory tract; as well as enough of samples to perform functional comparisons between PF and CU samples, but believe that these data is not enough to address differences in alpha- and beta-diversity between PF/CU. I find this argument a little vague and I strongly believe that the paper would benefit from including this information, but since it initially was just my suggestion to the authors, they are free to ignore it.
We would like to thank the reviewer's question. We consider that the sample number of PF is enough to explain the microbiota and the community function since the microbiota and its function were mainly dressed by the dominate species which could by fully explained by the data achieved from these samples. However, we claim that it is not enough to address differences in alpha- and beta-diversity between PF/CU because that the rarefaction curve of PF did not reach the saturation, while CU did. That means some species may still not been detected in the PF samples and this will cause the deviation of the bacterial alpha- and beta-diversity results.

Line 161, figure 3, the PF line is not far from reaching the plateu - it does seem that the line is approaching the asymptote, so i belive that 'far from saturation' is an overstatement and should be toned down.
We agree with the suggestion raised from the reviewer. After the double checking, we totally agreed with the reviewer and revised this statement to make it toned down (Line 160-162).

Reviewer #2: Still think that a quick read for English would be useful for this manuscript though the writing has improved. Examples of writing that needs correcting are as follows: Lines 34-35 in the abstract: "the vaginal microbiota plays important roles … " should be "role"; Line 91 "(the stringent selection rules …" should be "(for the stringent selection rules …"
We thank reviewer very much for the carefully reading and correction. We revised the manuscript according to the comments and checked the English carefully throughout the

manuscript.

The clustering process is still not described. Did the authors use hierarchical clustering, kmeans clustering …. What was the cutoff used to identify the clusters? Based on this and on figure Sup Fig 2 I don't believe that they achieved individual sub-cluster representation.
We thank reviewer for pointing out this information which should not be omitted. We applied centroid-linkage method for the hierarchical clustering in this study and the detailed information has been added in the methods (Line 194-195). After hierarchical clustering, we selected the samples with enough DNA amount in each sub-cluster as the representative candidates to do the further analysis.

Line 92 Supplementary Figure 2 does not provide details of the stringent selection rules.
We would like to thank the reviewer for the question. The reason why we cite Supplementary Fig. 2 here is one of the selection rules is according to the results of the clustering which presented in the Supplementary Fig. 2. However, as the reviewer pointed out, this figure did not provide the details of the stringent selection rules, so we deleted the citation in line 92.

Line 136 The authors should provide the reference to the "previous" study when they mention it.
We are grateful for the reviewer’s suggestion. We agreed with the reviewer and added the reference here, which is our recent study using 16S rRNA amplicon sequencing.

Close
