# Supplementary material for: The metagenome of the female upper reproductive tract
Source: Gigascience. 2018 Sep 6;7(10):giy107. doi: 10.1093/gigascience/giy107 (PMC6177736; doi:10.1093/gigascience/giy107)
Supplement: Reviewer_1_Report_(Original_Submission) -- Ekaterina Avershina — 6/12/2018 Reviewed [file giy107_reviewer_1_report_(original_submission).pdf]

Reviewer Report

Title: **The metagenome of the female upper reproductive tract**

Version: **Original Submission** **Date:** 6/12/2018

Reviewer name: **Ekaterina Avershina**

Reviewer Comments to Author:

The manuscript has vastly improved since the first submission, although some additional information and language editing is required.I would like to thank the authors for including more in-depth information about the functional data; i think p-values for the aforementioned differences in gene families have to be provided; maybe marking the significant differences on the figure. I also think that since this is actually the most novel data in the paper, the Supplementary Figure 3 should be moved to the main body of the paper.Authors have enough of samples to claim the gradient in microbiota over the reproductory tract; as well as enough of samples to perform functional comparisons between PF and CU samples, but believe that these data is not enough to address differences in alpha- and beta-diversity between PF/CU. I find this argument a little vague and I strongly believe that the paper would benefit from including this information, but since it initially was just my suggestion to the authors, they are free to ignore it. Line 161, figure 3, the PF line is not far from reaching the plateu - it does seem that the line is approaching the asymptote, so i belive that 'far from saturation' is an overstatement and should be toned down.

Level of Interest

Please indicate how interesting you found the manuscript: Choose an item.

Quality of Written English

Please indicate the quality of language in the manuscript: Choose an item.

Declaration of Competing Interests

Please complete a declaration of competing interests, considering the following questions:

- Have you in the past five years received reimbursements, fees, funding, or salary from an organisation that may in any way gain or lose financially from the publication of this manuscript, either now or in the future?
- Do you hold any stocks or shares in an organisation that may in any way gain or lose financially from the publication of this manuscript, either now or in the future?
- Do you hold or are you currently applying for any patents relating to the content of the manuscript?
- Have you received reimbursements, fees, funding, or salary from an organization that holds or has applied for patents relating to the content of the manuscript?
- Do you have any other financial competing interests?
- Do you have any non-financial competing interests in relation to this paper?

If you can answer no to all of the above, write 'I declare that I have no competing interests' below. If your reply is yes to any, please give details below.

I declare that I have no competing interests

I agree to the open peer review policy of the journal. I understand that my name will be included on my report to the authors and, if the manuscript is accepted for publication, my named report including any attachments I upload will be posted on the website along with the authors' responses. I agree for my report to be made available under an Open Access Creative Commons CC-BY license (http://creativecommons.org/licenses/by/4.0/). I understand that any comments which I do not wish to be included in my named report can be included as confidential comments to the editors, which will not be published.

I agree to the open peer review policy of the journal

To further support our reviewers, we have joined with Publons, where you can gain additional credit to further highlight your hard work (see: https://publons.com/journal/530/gigascience). On publication of this paper, your review will be automatically added to Publons, you can then choose whether or not to claim your Publons credit. I understand this statement. Yes
