# Supplementary material for: The metagenome of the female upper reproductive tract
Source: Gigascience. 2018 Sep 6;7(10):giy107. doi: 10.1093/gigascience/giy107 (PMC6177736; doi:10.1093/gigascience/giy107)
Supplement: Reviewer_2_Report_(Original_Submission) -- Zaid Abdo — 6/15/2018 Reviewed [file giy107_reviewer_2_report_(original_submission).pdf]

Reviewer Report

Title: The metagenome of the female upper reproductive tract

Version: Original Submission **Date:** 6/15/2018

Reviewer name: Zaid Abdo

Reviewer Comments to Author:

Still think that a quick read for English would be useful for this manuscript though the writing has improved. Examples of writing that needs correcting are as follows: Lines 34-35 in the abstract: "the vaginal microbiota plays important roles … " should be "role"; Line 91 "(the stringent selection rules …" should be "(for the stringent selection rules …"The clustering process is still not described. Did the authors use hierarchical clustering, kmeans clustering …. What was the cutoff used to identify the clusters? Based on this and on figure Sup Fig 2 I don't believe that they achieved individual sub-cluster representation.Line 92 Supplementary Figure 2 does not provide details of the stringent selection rules.Line 136 The authors should provide the reference to the "previous" study when they mention it.

Level of Interest

Please indicate how interesting you found the manuscript: Choose an item.

Quality of Written English

Please indicate the quality of language in the manuscript: Choose an item.

Declaration of Competing Interests

Please complete a declaration of competing interests, considering the following questions:

- Have you in the past five years received reimbursements, fees, funding, or salary from an organisation that may in any way gain or lose financially from the publication of this manuscript, either now or in the future?
- Do you hold any stocks or shares in an organisation that may in any way gain or lose financially from the publication of this manuscript, either now or in the future?
- Do you hold or are you currently applying for any patents relating to the content of the manuscript?
- Have you received reimbursements, fees, funding, or salary from an organization that holds or has applied for patents relating to the content of the manuscript?
- Do you have any other financial competing interests?
- Do you have any non-financial competing interests in relation to this paper?

If you can answer no to all of the above, write 'I declare that I have no competing interests' below. If your reply is yes to any, please give details below.

No to all above

I agree to the open peer review policy of the journal. I understand that my name will be included on my report to the authors and, if the manuscript is accepted for publication, my named report including any attachments I upload will be posted on the website along with the authors' responses. I agree for my report to be made available under an Open Access Creative Commons CC-BY license (http://creativecommons.org/licenses/by/4.0/). I understand that any comments which I do not wish to be included in my named report can be included as confidential comments to the editors, which will not be published.

I agree to the open peer review policy of the journal

To further support our reviewers, we have joined with Publons, where you can gain additional credit to further highlight your hard work (see: https://publons.com/journal/530/gigascience). On publication of this paper, your review will be automatically added to Publons, you can then choose whether or not to claim your Publons credit. I understand this statement. Yes
